# Childhood Leukemia Survival in the US-Mexico Border: Building Sustainable Leukemia Care Using Health Systems Strengthening Models

**DOI:** 10.1200/GO.23.00123

**Published:** 2023-06-03

**Authors:** Paula Aristizabal, Rebeca Rivera-Gomez, Andrew Chang, Mario Ornelas, Maribel Ramirez, Gabriela Tamayo, Angelica Martinez, Raul C. Ribeiro, William Roberts

**Affiliations:** ^1^ Peckham Center for Cancer and Blood Disorders, Rady Children's Hospital San Diego, San Diego, CA; ^2^ Department of Pediatrics, Division of Pediatric Hematology/Oncology, University of California San Diego, La Jolla, CA; ^3^ Population Sciences, Disparities and Community Engagement, University of California San Diego Moores Cancer Center, La Jolla, CA; ^4^ University of California San Diego Altman Clinical and Translational Research Institute, Dissemination and Implementation Science Center, La Jolla, CA; ^5^ Hospital General de Tijuana, Universidad Autónoma de Baja California, Tijuana, Mexico; ^6^ School of Medicine, University of California San Diego, La Jolla, CA; ^7^ Department of Oncology, Leukemia/Lymphoma Division, and Global Pediatric Medicine, St Jude Children's Research Hospital, Memphis, TN

## Abstract

**PURPOSE:**

Pediatric leukemia outcomes are poor in most low- and middle-income countries (LMICs) and exacerbated by health care systems ill equipped to manage cancer. Effective leukemia management in LMICs involves curating epidemiologic data; providing health care workforce specialty training; developing evidence-based treatments and supportive care programs; safeguarding access to medications and equipment; providing patient and family psychosocial, financial, and nutritional support; partnering with nongovernmental organizations, and ensuring treatment adherence.

**METHODS:**

In 2013, through a partnership between North-American and Mexican institutions, we used the WHO *Framework for Action*, a health systems strengthening model to implement a leukemia care sustainable program aimed at improving acute lymphoblastic leukemia (ALL) outcomes at a public hospital in Mexico. We prospectively assessed clinical features, risk classification, and survival outcomes in children with ALL at Hospital General-Tijuana from 2008 to 2012 (preimplementation) and from 2013 to 2017 (postimplementation). We also evaluated program sustainability indicators.

**RESULTS:**

Our approach led to a fully-staffed leukemia service, sustainable training programs, evidence-based and data-driven projects to improve clinical outcomes, and funding for medications, supplies, and personnel through local partnerships. Preimplementation and postimplementation 5-year overall survival for the entire cohort of children with ALL, children with standard-risk ALL, and children with high-risk ALL improved from 59% to 65% (*P* = .023), 73% to 100% (*P* < .001), and 48% to 55% (*P* = .031), respectively. All sustainability indicators improved between 2013 and 2017.

**CONCLUSION:**

Using the health systems strengthening WHO *Framework for Action* model, we improved leukemia care and survival in a public hospital in Mexico across the US-Mexico border. We provide a model for the development of similar programs in LMICs to sustainably improve leukemia and other cancer outcomes.

## INTRODUCTION

Despite significant strides in curative treatments made over the past 4 decades, acute lymphoblastic leukemia (ALL) remains one of the leading causes of childhood death and morbidity after infancy.^1^ In high-income countries (HICs), 5-year overall survival (OS) ranges from 80% to 90%. This dramatic improvement has been attributed to advancements in diagnostic technologies, risk-stratification systems, and treatment protocols driven by cooperative clinical trials.^2,3^ Nevertheless, ALL survival outcomes have not been equitable around the world. As the global pediatric cancer burden grows in low- and middle-income countries (LMICs),^4-6^ ALL OS in LMICs has been reported between 10% and 60%.^7,8^ These disparate outcomes could be mitigated with wider availability of critical, specialized, and supportive care.^5,6,9^

**CONTEXT**

**Key Objective**
To describe the application of the health systems strengthening WHO *Framework for Action* to a twinning program between Rady Children's Hospital San Diego, USA, and Hospital General-Tijuana, Mexico, to evaluate its impact on capacity building for high-quality, sustainable care and on improving survival for children with leukemia in a low- and middle-income country (LMIC) setting.
**Knowledge Generated**
Our initiative resulted in a fully staffed pediatric leukemia service with protocols, organization, and structure designed to address each of the six building blocks of the WHO *Framework for Action* model. Preimplementation and postimplementation results demonstrate significant improvements in survival in children with leukemia in Baja California, Mexico, and improved program sustainability indicators.
**Relevance**
The success of the application of the WHO *Framework for Action* model to our twinning program supports the incorporation of the six building blocks when developing pediatric leukemia and other cancer control programs in LMICs.

In Mexico, ALL is the most common pediatric cancer and the second leading cause of death between age 5 and 14 years^10^ with OS reported to be 20%-40%.^10-12^ State of the art diagnostic and clinical services, access to pediatric oncologists, and specialized and critical care training for all providers are limited in many Mexican institutions.^13^ To mitigate these disparities in access and outcomes, we established in 2008 a twinning program between Rady Children's Hospital San Diego, St Jude Children's Research Hospital, and Hospital General-Tijuana (HGT), Mexico, in the US-Mexico border region.^6,14^ Although this model improved access to care and clinical outcomes at HGT from 2008 to 2013, its dependency on the institutions in HIC required an alternative strategy to ensure long-term sustainability and independence. To address this, in 2013, we used the WHO health systems strengthening *Framework for Action* model^15^ to implement measures to decrease the dependency of the twinning program on external resources, promote sustainability, and continue to improve ALL outcomes in the public sector in Mexico. We describe the developmental milestones on the basis of the WHO *Framework for Action* and its early impact on clinical outcomes for patients with ALL at HGT preimplementation from 2008 to 2012 and postimplementation from 2013 to 2017. We also report indicators of sustainability in 2013 and 2017.

## METHODS

### Context and Health Systems Strengthening Model for Developing High-quality Leukemia Care

HGT is a major public referral hospital in northwestern Mexico, serving approximately three million people. Yet, in 2008, the hospital had limited treatment options, and outcomes for children with cancer were dismal in Tijuana and the state of Baja California. HGT had neither a dedicated pediatric oncology unit nor a pediatric intensive care unit. There were no pediatric oncologists or pediatric oncology nurses. Diagnostic equipment was severely limited, and stock outs of medications were frequent. Responding to the need for high-quality pediatric cancer care in the US-Mexico border region, Rady Children's Hospital San Diego in partnership with St Jude Children's Research Hospital launched a collaborating twinning program at HGT in 2008.^6,14^ *Twinning* involves a partnership between pediatric cancer centers in HICs and LMICs aimed at improving survival and other clinical outcomes by sharing knowledge, expertise, and resources.

**FIG 1:**
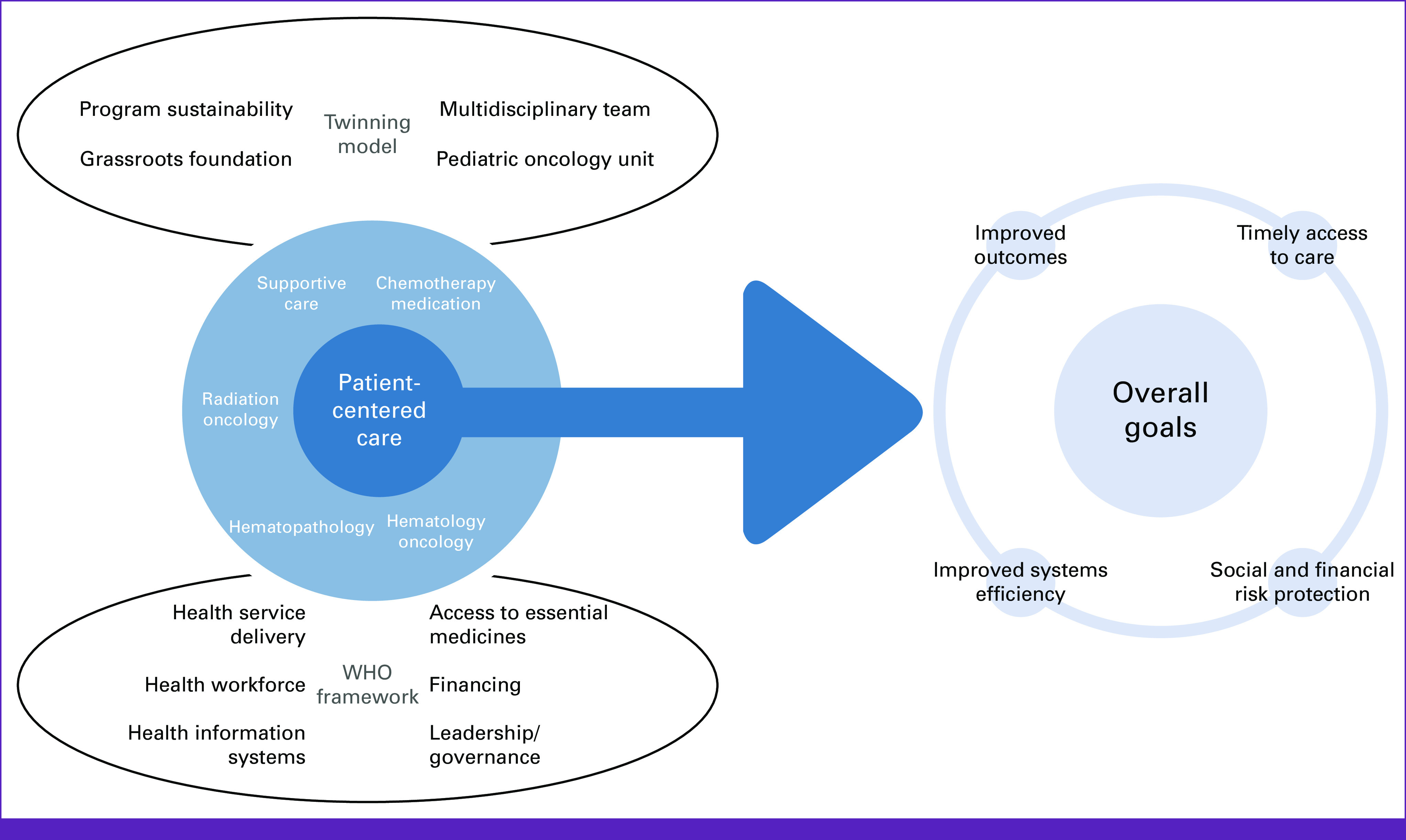
Twinning-WHO framework combination model to achieve high-quality leukemia care.

As the number of patients with ALL increased and the needs escalated, we adopted the health systems strengthening WHO *Framework for Action*^15^ model in 2013 as a development roadmap to implement an independent and sustainable leukemia care program. The building blocks of the WHO *Framework for Action* describe essential and interdependent functions necessary for the optimal performance of a health system and include service delivery, workforce, information systems, access to essential medicines, financing, and local leadership/governance. We used the WHO *Framework for Action* as a guide in setting substantive and comprehensive milestones necessary for developing sustainable capacity for high-quality pediatric leukemia services (Fig 1).

### Leukemia Care Program Implementation: Application of the WHO Framework for Action Model

We conducted a needs assessment by using an instrument adapted from St Jude Children's Research Hospital's needs assessment tools^14^ and developed and set a 10-year action plan with objectives outlined according to the WHO *Framework for Action*.

#### *Block 1. Health Service Delivery*.

To ensure the delivery of effective, safe, and high-quality clinical care with minimum waste, we focused on four main areas.

##### Infrastructure.

Our first objective was to increase capacity through pediatric leukemia-specific infrastructure within the dedicated Pediatric Hematology Oncology Unit (PHOU) inaugurated in 2008 at HGT.

##### Clinical monitoring and supportive care.

To enhance the ability to monitor patient status and respond to clinical needs, we introduced the Pediatric Early Warning Score (PEWS) system.^16^ We secured a Mexican federal grant, developed the PEWS team, and trained all PHOU staff according to St Jude Children's Research Hospital PEWS implementation guidelines. Additionally, the *Golden Hour* project was established to ensure faster antibiotic initiation during neutropenic fever events to reduce sepsis risk.^17,18^ HGT staff developed a systematized protocol for supply inventory, indication, and administration of antibiotics.

##### ALL treatment.

Led through bimonthly meetings with a Spanish-speaking pediatric hematologist/oncologist (P.A.) with expertise in pediatric leukemia from Rady Children's Hospital San Diego, our team initiated an action plan to establish HGT's first locally adapted chemotherapy protocol^19^ and guidelines for blood products transfusions adapted to resource-limited settings, created training materials and milestones for assessing the impact, and established a weekly leukemia board to identify areas of improvement related to management of ALL. We also began the Project MEJOR (better in English), a protocol for applying a comprehensive and system-by-system treatment plan for each patient with ALL in the PHOU during daily rounds.

##### Treatment adherence.

To address treatment abandonment, we initiated projects to improve adherence to treatment, particularly during the 1- to 2-year long ALL maintenance chemotherapy. Nurses led education initiatives for all patients with ALL and their caregivers.

#### *Block 2. Health Workforce*.

We focused our training and education efforts on diagnostics and specialized outpatient and inpatient care for critically ill patients with ALL. We appointed a lead pediatrician and two nurse educators at HGT to develop a primarily on-site training in-person curriculum in pediatric leukemia for physicians, nurses, and allied staff.

Implementation of the leukemia care program included multidisciplinary daily rounds, a weekly leukemia board, and 24/7 coverage in the inpatient unit by trained pediatricians. We recruited six pediatric oncologists and eight pediatricians over 5 years. Additionally, skill development and upgraded equipment for the pathology and hematology laboratories were emphasized as early priorities.

To build the PHOU's nursing workforce, we recruited nursing staff permanently assigned to the PHOU. The nurse educators taught a 6-week orientation course developed by St Jude Children's Research Hospital,^20^ including advanced pediatric leukemia clinical competencies. Training was supplemented by visits to Rady Children's Hospital San Diego and to St Jude Children's Research Hospital affiliated sites in Guatemala and Chile.^20^

#### *Block 3. Health Information Systems*.

We aimed to establish a system for the collection, analysis, application, and dissemination of reliable data to monitor clinical progress and effectively design data-driven quality improvement projects. To achieve these goals, we developed a hospital-based cancer registry at HGT and partnered with the Tijuana population-based cancer registry, BajaREG.^21^

#### *Block 4. Access to Essential Medicines*.

To ensure a consistent supply of equipment, medications, and supplies with priority placed on quality, safety, efficacy, and cost-effectiveness, we aimed to develop practices to reduce waste and garnered financial support from the HGT leadership and Patronato, a local grassroots nongovernmental foundation.

#### *Block 5. Financing*.

To address financial sustainability of ALL management, we applied for national accreditation to ensure funding through the Popular Insurance Program (Seguro Popular)^22^ and established a partnership with Patronato. Additional startup funding was obtained from Rady Children's Hospital San Diego and St Jude Children's Research Hospital.

#### *Block 6. Leadership/Governance*.

Our partnership model between Rady Children's Hospital San Diego and HGT was aimed at promoting a bilateral commitment of time, personnel, and financial resources as the basis for collaboration to improve leukemia care. An early priority included forming a multidisciplinary implementation team to engage key stakeholders in vision setting, with an emphasis on establishing a local governance structure, accountability, and transparency at the individual and organizational levels, and to ensure long-term sustainability. Rady Children's Hospital San Diego and St Jude Children's Research Hospital provided leadership training for key staff at HGT.

### ALL Patient Outcome Measures

We prospectively collected data on children younger than 18 years with ALL diagnosed between 2008 and 2017 at HGT. Risk stratification was applied to all patients using the National Cancer Institute risk classification criteria, immunophenotyping, genetic features, and measurable residual disease (Table 1). Two cohorts were analyzed separately: preprogram implementation from 2008 to 2012 and postimplementation from 2013 to 2017. Outcomes including OS and event-free survival (EFS) were estimated and stratified according to risk of relapse (standard-risk [SR] or high-risk [HR]) and compared between the two cohorts. Events included relapse, treatment abandonment (interruption of treatment of at least 4 weeks for nonmedical reasons), and death. The Institutional Review Boards for the University of California San Diego/Rady Children's Hospital San Diego and HGT approved this study. Informed consent was obtained from parents/legal guardians.

**TABLE 1:**
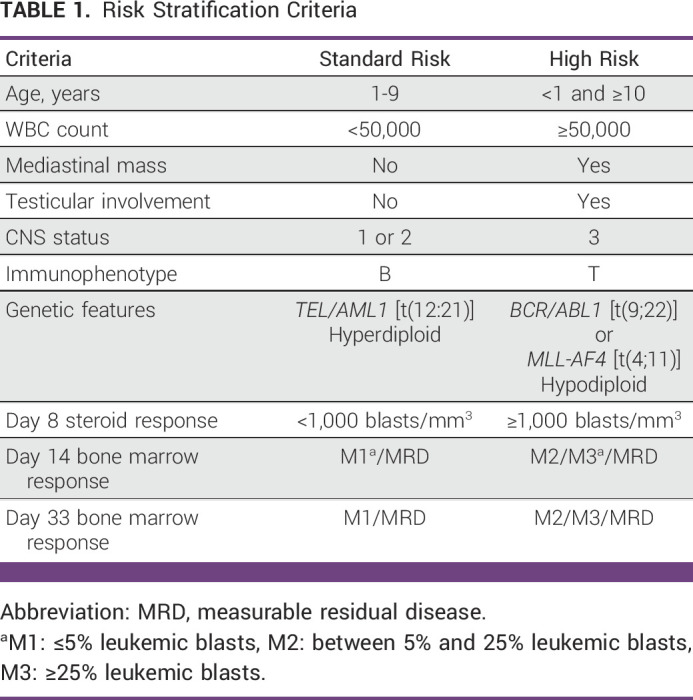
Risk Stratification Criteria

### Statistical Analysis

We performed descriptive statistics to describe baseline patient characteristics (age, sex, and risk stratification). Kaplan-Meier survival curves were generated for OS and EFS, and hazard ratios and 95% CIs were calculated. Log-rank tests were used to determine the statistical significance of differences in OS and EFS between the preimplementation and the postimplementation cohorts. Statistical analyses were conducted using R software (3.5.0; R Foundation for Statistical Computing, Vienna, Austria). We considered a *P* ≤ .05 to indicate statistical significance.

### Program Sustainability Assessment

Sustainability indicators encompassed three major domains: process, staff, and organization and were analyzed in 2013 and at the end of 2017 using a validated score-based sustainability tool.^23^ The National Health Service (NHS) Sustainability Model is a self-assessment tool detailing 10 key indicators that increase the likelihood of sustainability and continuous improvement for a specific change that has been introduced into an organization. Scores represented leaders' and point-of-care staff's perceptions.

## RESULTS

### WHO Framework for Action Implementation Results

The results of the implementation of the WHO *Framework for Action* model are summarized in Table 2.

**TABLE 2:**
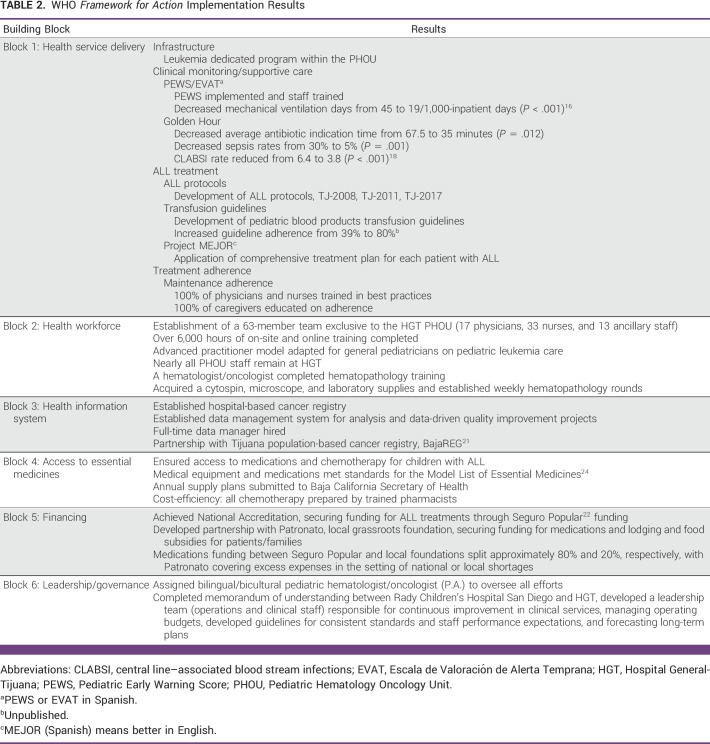
WHO *Framework for Action* Implementation Results

### ALL Clinical Outcomes

Approximately 20-22 children with ALL per year were expected in Tijuana on the basis of the childhood population. The majority (approximately 60%) were diagnosed and received care at HGT.

In total, 109 children with newly diagnosed ALL were included in this study and were divided into two cohorts: preimplementation (n = 49, from 2008 to 2012) and postimplementation (n = 60, from 2013 to 2017). Patient characteristics are described in Table 3.

**TABLE 3:**
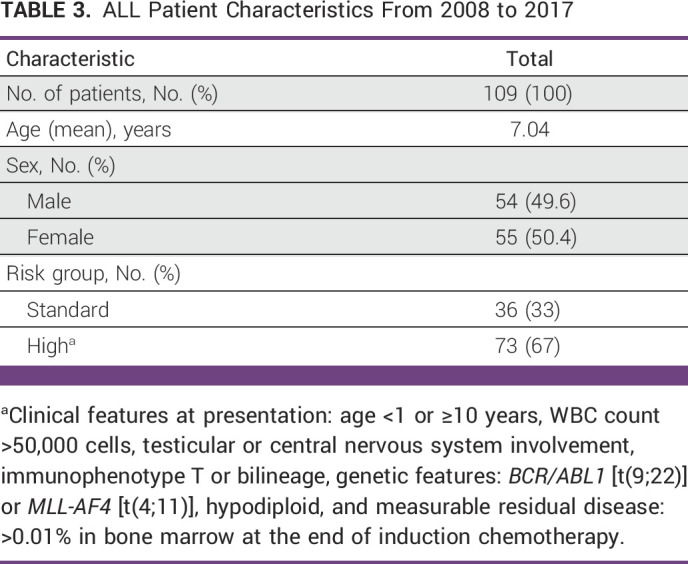
ALL Patient Characteristics From 2008 to 2017

**FIG 2:**
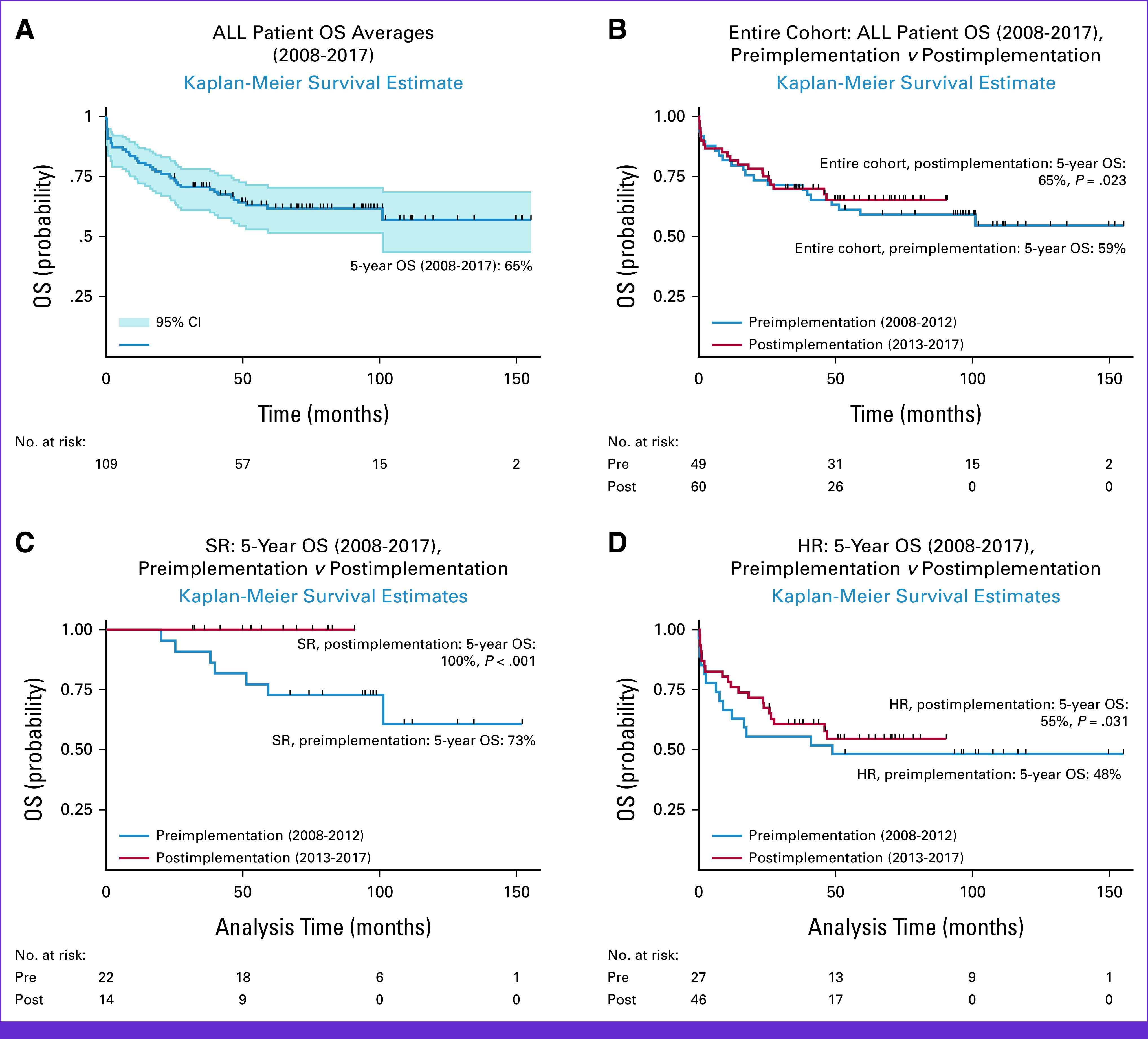
ALL survival outcomes. HR, high risk; OS, overall survival; SR, standard risk.

Five-year OS for the entire cohort was 65% in 2017, and 5-year EFS was 56% (Fig 2A). Among the entire cohort, between preprogram implementation and postimplementation, 5-year OS for children with ALL improved from 59% to 65% (*P* = .023; Fig 2B). Additionally, preimplementation and postimplementation 5-year OS for children with SR ALL improved from 73% to 100% (*P* < .001), and for children with HR ALL, OS improved from 48% to 55% (*P* = .031; Figs 2C and 2D).

### Sustainability Assessment Results

**FIG 3:**
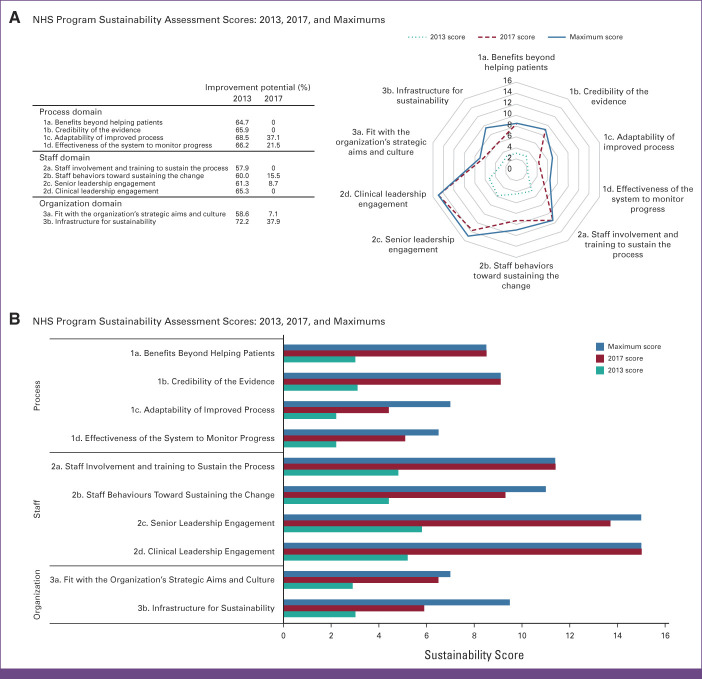
Program sustainability assessment. NHS, National Health Service.

All the sustainability indicators assessed had an improvement potential when documented for process, staff, and organization domains (Fig 3). The two indicators of the process domain with the highest improvement potential were the *adaptability of improved processes* and the *effectiveness of the system to monitor progress*. *Clinical leadership engagement* was considered the most critical indicator in the staff domain. In the organization domain, the *infrastructure for sustainability* indicator was the most important (Fig 3). In 2017, 4 of 10 sustainability indicators reached the maximum improvement potential, 2 of 10 sustainability indicators had improvement potentials below 10%, and the remaining 4 of 10 sustainability indicators had improvement potentials below 40% (Fig 3).

## DISCUSSION

The application of the WHO's health systems strengthening *Framework for Action* model and its six building blocks: service delivery, workforce, information systems, access to essential medicines, financing, and leadership/governance resulted in the successful implementation of an effective and sustainable leukemia care program in the US-Mexico border region. The 5-year OS and EFS improved significantly postprogram implementation. Moreover, we demonstrated improvement across all three NHS sustainability domains: process, staff, and organization. These results support that pediatric ALL outcomes in LMIC settings can be meaningfully improved through partnership programs designed according to health systems strengthening models.

The efficacy in improving ALL and other pediatric cancer outcomes through twinning programs has been well documented across diverse LMIC settings including in Latin America, the Middle East, and East Asia.^25-27^ However, ALL outcomes, particularly survival, still lag behind those in HICs.^2-4^ Although causes of failure can vary across LMICs, the application of health systems strengthening models focused on building sustainability are essential to closing this survival gap. According to our sustainability assessment, improvement potential was achieved in 2013 after implementing the leukemia program at HGT and sustained into 2017 in 6 of the 10 sustainability indicators, enabling greater capitalization of opportunities to improve survival outcomes. These improvements in sustainability suggest that the improved clinical outcomes can be sustained long-term and allow the program to adjust and continue to make improvements to respond to new challenges in providing comprehensive leukemia care in resource-constrained settings.

A significant improvement in OS and EFS between preprogram and postprogram implementation for both patients with SR and HR ALL and a steadily increasing 5-year OS rate of 65% for the entire ALL cohort within a decade of our program's inception demonstrate sustained high-quality care and improved outcomes. Additionally, an increase of more than 30% improvement potential across all 10 sustainability indicators from 2013 to 2017 suggests the success of the WHO *Framework for Action* approach. The WHO *Framework for Action* model and its focus on long-term sustainability and a comprehensive approach to program implementation enabled us to systematically address commonly recognized elements to improving ALL outcomes in LMICs, including specialized training,^4-6^ development of locally adapted treatment protocols,^19^ providing supportive care and infection control,^9,17,18,28,29^ ensuring funding for treatment,^6,14,22,25^ supporting treatment adherence, and reducing financial toxicity toward affected patients' families through lodging and food subsidies.^6-8,14^

The scale of our model is small; nevertheless, there are precedents for endeavoring toward large-scale pediatric leukemia care improvements in LMICs. Examples include the Asociación de Hemato-Oncología Pediátrica de Centro América and Mexico's *Seguro Popular* initiative, which in 2008 introduced new coverage for pediatric cancer treatment. However, Mexico still faces challenges in providing effective ALL treatment. It was estimated that only 48% of eligible patients were covered by *Seguro Popular*, and clinical outcomes remain variable across different regions.^22^ For instance, studies report that 62% of pediatric oncologists in Mexico remain concentrated in its three largest cities (Mexico City, Guadalajara, and Monterrey).^22^ Although two studies^11,30^ report a higher prevalence (58%-78%) of HR ALL, similar to our rate of 67%, a lack of a Mexican national cancer registry^21^ limits the epidemiological understanding of pediatric ALL in Mexico. Therefore, there is an urgent need to implement more evidence-based pediatric leukemia care through sustainable national collaborative efforts.

Our study has limitations including a small sample size in a large public reference hospital that precludes in-depth analysis and generalizability to other settings. Additionally, a lack of a hospital-based cancer registry before the twinning cancer program implementation in 2008 at HGT limited our ability to accurately compare survival rates with those from before the twinning program started. Furthermore, since risk categorization is subjected to advancements in diagnostic technologies and expertise gained by the treatment team over time, especially when using criteria such as molecular classification and measurable residual disease, potential risk migration during the postimplementation period may have altered accuracy of survival data when stratifying by risk groups. Regarding reproducibility, our leukemia care program is unique in that it benefited from the proximity (24 miles) between Rady Children's Hospital San Diego and HGT, enabling a large degree of in-person collaboration that is uncommon to most global health partnerships. Finally, our results are limited to the period when Seguro Popular was in place. Seguro Popular is in the process of transitioning to a new system Instituto de Salud para el Bienestar (INSABI), which aims for universal coverage in Mexico. Thus, our model and results may not be translated over to private or social security settings and sustainability would need to be remeasured after INSABI is fully implemented in Baja California.

In conclusion, the WHO *Framework for Action* encompassing approach to its six building blocks was successfully incorporated into an existing twinning model to improve survival in children with ALL in an effective and sustainable way in the US-Mexico border. This model appeals to broader impact in Mexico, with priorities concerning healthcare workforce and delivery standards, building national population-based cancer registries, improving national funding mechanisms, and facilitating collaboration between public and private organizations across regions. Improved pediatric leukemia survival outcomes are achievable in LMICs through international partnerships that apply health systems strengthening models to build capacity and sustainability. Our model serves as an example for future global partnerships aimed to establish pediatric and adult cancer services in LMICs. Interdisciplinary, concerted, and cooperative approaches improve the likelihood of successful collaborative projects. Forthcoming initiatives should consider the implementation of standardized approaches for data collection through cancer registries and surveillance systems, the building of sustainability through financial and bidirectional leadership structures and the provision of patient-centered care to mitigate socioeconomic barriers to optimal clinical outcomes. Future research should evaluate best practices in establishing global health partnerships aimed at improving cancer care through the application of health systems strengthening models adapted to local health systems in each unique community.
